# Click Conjugation of Boron Dipyrromethene (BODIPY) Fluorophores to EGFR-Targeting Linear and Cyclic Peptides

**DOI:** 10.3390/molecules26030593

**Published:** 2021-01-23

**Authors:** Tyrslai M. Williams, Nichole E. M. Kaufman, Zehua Zhou, Sitanshu S. Singh, Seetharama D. Jois, Maria da Graça H. Vicente

**Affiliations:** 1 Department of Chemistry, Louisiana State University, Baton Rouge, LA 70803, USA; twil161@lsu.edu (T.M.W.); nkaufm1@lsu.edu (N.E.M.K.); zzhou2@lsu.edu (Z.Z.); 2 School of Basic Pharmaceutical and Toxicological Sciences, College of Pharmacy, University of Louisiana at Monroe, Monroe, LA 71201, USA; singhss@warhawks.ulm.edu (S.S.S.); jois@ulm.edu (S.D.J.)

**Keywords:** BODIPY, EGFR, peptide, click reaction

## Abstract

Through a simple 1,3-cycloaddition reaction, three BODIPY-peptide conjugates that target the extracellular domain of the epidermal growth factor receptor (EGFR) were prepared and their ability for binding to EGFR was investigated. The peptide ligands K(N_3_)LARLLT and its cyclic analog cyclo(K(N_3_)larllt, previously shown to have high affinity for binding to the extracellular domain of EGFR, were conjugated to alkynyl-functionalized BODIPY dyes **1** and **2** via a copper-catalyzed click reaction. This reaction produced conjugates **3**, **4**, and **5** in high yields (70–82%). In vitro studies using human carcinoma HEp2 cells that overexpress EGFR demonstrated high cellular uptake, particularly for the cyclic peptide conjugate **5**, and low cytotoxicity in light (~1 J·cm^−2^) and darkness. Surface plasmon resonance (SPR) results show binding affinity of the three BODIPY-peptide conjugates for EGFR, particularly for **5** bearing the cyclic peptide. Competitive binding studies using three cell lines with different expressions of EGFR show that **5** binds specifically to EGFR-overexpressing colon cancer cells. Among the three conjugates, **5** bearing the cyclic peptide exhibited the highest affinity for binding to the EGFR protein.

## 1. Introduction

The study of protein–protein interactions (PPIs) is very useful when investigating mutagenic behaviors that lead to diseases and oncogenic mutations [1,2,3]. Several studies offer insight on the behavior of PPIs in human diseases, in particularly oncogenic mutations of the epidermal growth factor receptor (EGFR) in colorectal cancer (CRC) [4]. This mutation makes targeting EGFR for CRC detection very attractive. To capitalize on this strategy, recent studies have targeted EGFR, as a method to increase tumor cell specificity for cancer therapy [4,5,6,7]. Practices including FDA-approved anti-EGFR antibody cetuximab [8,9], single-chain anti-EGFR ScFvEGFR [10,11,12], anti-EGFR affibody [13,14], accessible small peptides [15], and tyrosine kinase inhibitors [16,17] have all been employed to understand targeting EGFR.

EGFR is a ligand-stimulated receptor that holds a crucial role in regulating cellular functions, more directly comprising cell proliferation and survival [4,5,6,7,18]. In addition, EGFR houses a single polypeptide backbone chain that is understood to be an important component in exploring effective treatments and screening of cancers, such as CRC. While the overexpression of EGFR on the surface of CRC is due to mutations in the gene, and its upregulation, the activation of EGFR can also occur through mutation in the kinase domain [17,18].

While current research has identified several practices for targeting EGFR, our group has previously prepared and investigated EGFR-targeted porphyrin-peptide [19], phthalocyanine-peptide [20], and BODIPY-peptide conjugates [21,22,23]. In those studies, two small peptides reported in the literature, designated EGFR-L1 (LARLLT) [24] and EGFR-L2 (YHWYGYTPQNVI) [25], showed to have high EGFR specificity both in in vitro and in vivo studies, were used. The conjugates linked to EGFR-L1 via a short PEG linker showed enhanced water solubility when compared with the conjugates attached to EGFR-L2, which bears a longer hydrophobic peptide sequence [20,21]. Additionally, these peptide-bearing conjugates showed enhanced EGFR-targeting ability, and up to 90-fold increased accumulation in EGFR-overexpressed cells compared with unconjugated fluorophore [21]. These results suggest that fluorophores conjugated to EGFR-L1 and EGFR-L2 have substantially increased EGFR- targeting ability and may be very useful for the early detection and diagnosis of CRC and other EGFR-overexpressing cancers. While EGFR-L1 is known to bind in domain I of EGFR, away from the EGF binding pocket, EGFR-L2 binds to the EGF binding pocket on the extracellular domain of EGFR. Furthermore, previous studies suggest that EGFR-L1 conjugates tend to bind to EGFR with higher affinity compared with the EGFR-L2-based conjugates [19,20,26]. This might be a result of EGFR-L1 binding away from the EGF binding pocket, allowing for interactions with various conformations of EGFR, as the binding site for EGFR-L1 in domain I is not affected by conformational changes in EGFR [19].

Designing a fluorophore that is robust enough to handle the biological framework of CRC requires the scaffold to be robust, easily assembled, and visible enough to ensure proper screening. One must consider the development of these types of compounds to ensure stability and biocompatibility. Peptides bear an amine or carboxyl terminus available for conjugation to fluorophores such as porphyrins, phthalocyanines and boron dipyrromethenes (BODIPYs). BODIPYs are extremely useful fluorophores because of their tunable core structure and excellent photophysical properties [27,28,29]. Research shows that BODIPYs maintain a highly spectroscopic tunable core that is stable under various conditions. Increasing efficient approaches for conjugating BODIPY dyes to peptides requires that a clear position for attachment be available at each molecule. In peptide synthesis, the most evident attachment point is the exposed free amino group at the N-terminus of the sequence that can be used in traditional conjugation through amidation. Over time research has recognized the simplicity in forming attachment points during peptide synthesis (amines), however, the required carboxyl component on BODIPY dyes is not as easily attained, and the conjugation yields are moderate to low [23]. Therefore, a simple and high-yielding conjugation method, such as a so-called “click” reaction [30,31] has become more attractive in the last decade.

The copper(I)-catalyzed azide-alkyne cycloadditions, or click reactions, have a multitude of suitable features, including mild reaction conditions, short completion times, simple to no purification required, and high stability of the resulting triazoles under various reaction conditions, such as to air and moisture. Because of these benefits, the 1,3-dipolar cycloaddition is very attractive to researchers performing porphyrinoid conjugations for use in imaging and photodynamic therapy [32,33,34,35]. Even more striking is that this cycloaddition is especially fitting for sensitive constituents such as peptides, carbohydrates, and nucleotides. The overall attraction of click chemistry is deep-rooted in its nature to be selective, mild, and adaptable [36,37,38]. Other key components of click reactions lie in their ability to be nearly quantitative through the robust behavior and insensitivity of the resulting triazoles to various conditions [37]. The orthogonal ligation that occurs is suitable for various biomolecular pairing and in vivo labeling. The 1,2,3-triazoles formed in this reaction are stable under acidic and basic conditions, reductive and oxidative environments, and are resistant to proteolytic cleavages [38]. In addition, triazoles are also resilient to metabolic degradation. Overall, the steady nature of click compounds creates an ideal platform for developing stable fluorophore conjugates for bioimaging applications. 

Previous studies report the use of BODIPY dyes as the fluorescent component in cell-targeted conjugates bearing various targeting moieties for use in in vivo imaging. For example, Wolfbeis et al. [39] reported fluorescently labeled biomolecules through the use of copper-free and copper-mediated click chemistry. Wolfbeis et al. designed clickable BODIPY fluorophores for labeling of the azide-modified surface glycans of CHO cells. Akkaya et al. [40] used click chemistry in the synthesis of a bay region BODIPY dye attached to a perylenediimide (PDI). Akkaya et al. envisioned the molecule suitable for light harvesting which allows a large cross-section for the absorption of visible light. These studies were able to show that the excitation energy was efficiently channeled to the PDI core. Overkleeft et al. [41] designed three acetylene-functionalized BODIPY dyes to identify an azido-bearing epoxomicin analogue by fluorescence. Overkleeft et al. performed the functionalization through the Huisgen 1,3-dipolar cycloaddition, which yielded a group of fluorescent epoxomicin-derived proteasome probes. 

We have previously reported the conjugation of BODIPYs to the EGFR-L1 peptide via traditional amidation reactions [23], and nucleophilic additions to isothiocyanato-functionalized fluorophores [21,22]. Herein we report the use of click chemistry for conjugation of two EGFR-L1-derived peptides bearing an azide N-terminus (cycloL1.1 and L1.5) [26] to alkynyl-functionalized BODIPYs. The use of click chemistry allowed the employment of mild reaction conditions that are compatible with the BODIPY and peptide scaffolds, increasing the conjugation yield over alternative methodologies, and simplifying conjugate purification. We have previously used click chemistry to conjugate low molecular weight PEG groups and carbohydrates to BODIPYs, for enhanced aqueous solubility and cellular permeability [42]. Furthermore, to increase the stability of peptides in vivo we introduced conformational and configurational constraints, including sequence cyclization and change in the chirality of amino acids. The structure-activity investigation of the EGFR-L1 peptide with key modifications in its structure is described in our earlier report [26]. The modified peptide L1.5 exhibited increased affinity for EGFR compared to the parent peptide, while cycloL1.1 also exhibited enhanced stability in human serum. Herein we report the synthesis of two BODIPYs bearing an alkynyl handle for conjugation via an azide-functionalized lysine side chain to two EGFR-L1 peptide derivatives that have shown increased EGFR binding affinity and stability relative to EGFR-L1.

## 2. Results and Discussion

### 2.1. Synthesis

**Scheme 1:**
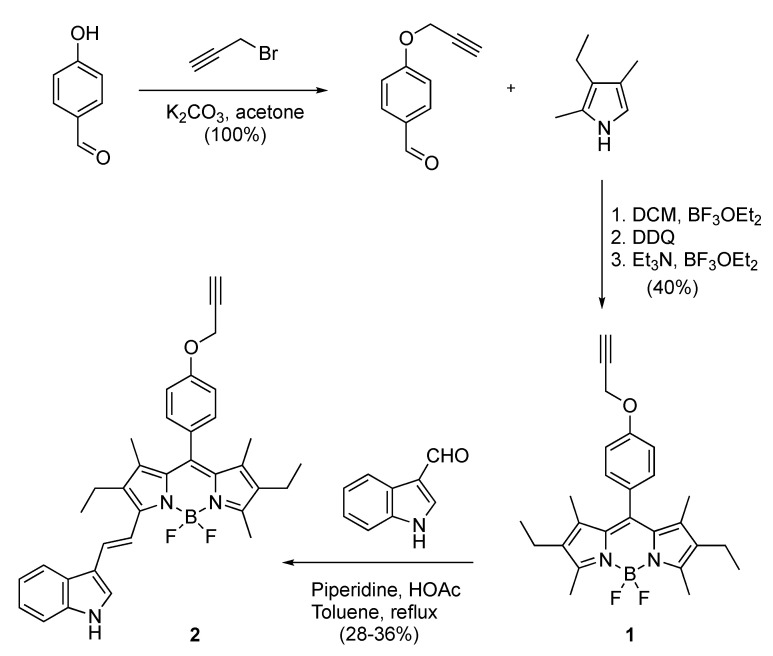
Synthetic route to alkynyl-functionalized BODIPYs **1** and **2**.

The alkynyl-functionalized BODIPYs **1** and **2** used in the conjugation reactions were synthesized as we have previously reported [42]. In brief, *p*-propargyloxy-benzaldehyde reacted with 3-ethyl-2,4-dimethylpyrrole in the presence of BF_3_·OEt_2_, followed by DDQ oxidation and boron complexation, to afford BODIPY **1** in 40% yield (see Supporting Information). Near-IR absorbing and emitting BODIPY **2** was prepared from **1** using indole-3-carbaldehyde in a Knoevenagel condensation [43]. Styryl-derived BODIPY compounds exhibit significant red-shifted fluorescence emissions due to the extension of the π-conjugation (Scheme 1). Additionally, the incorporation of an indole moiety has been observed to induce enhanced solubility and cellular permeability, possibly due to its resemblance to tryptophan. Tryptophan is a naturally occurring amino acid and an important component of many enzymes and proteins [44]. Therefore, the attachment of a single indolyl styryl group to the BODIPYs’ core results in an asymmetric mono-styryl BODIPY that displays a ca. 90 nm bathochromic shift on its absorption and emission profiles, and also enhanced solubility, cellular permeability, and overall biological efficacy [42,45]. 

The Knoevenagel reaction of BODIPY **1** in the presence of indole-3-carbaldehyde, piperidine, and glacial acetic acid in refluxing toluene, using a Dean-Stark apparatus, afforded the mono-styryl BODIPY **2** in 28–36% yields, due to the concomitant formation of the distyryl derivative. 

**Figure 1:**
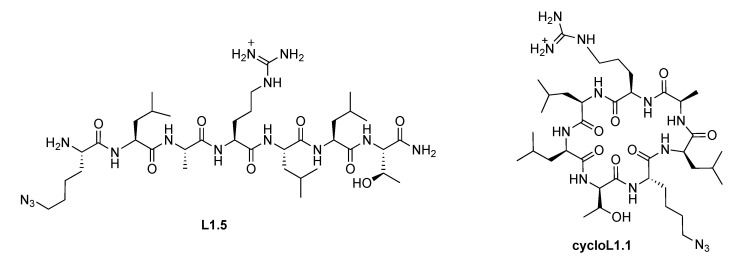
Azido peptides used for conjugation to BODIPYs **1** and **2**.

The EGFR-targeting peptide ligand EGFR-L1 (LARLLT) was reported in 2009 by Song et al. [24] and showed to have high affinity for the extracellular domain of EGFR, both in vitro and in vivo. In previous work, we reported the conjugation of this peptide to a porphyrin [19], phthalocyanine [20], and BODIPY dyes [21,22,23], using either traditional amidation methodologies or nucleophilic addition to isothiocyanato-functionalized fluorophores. These studies indicated that the mode of conjugation significantly influences the binding affinity of the resulting conjugates to EGFR. In order to conjugate EGFR-L1 to BODIPYs **1** and **2** using click chemistry, we modified the EGFR-L1 peptide via the introduction of an azide-functionalized lysine residue, to form L1.5 (K(N_3_)LARLLT) (Figure 1) [26]. In addition, to improve the stability of the L1.5 peptide in vivo, we performed a cyclization reaction to afford cycloL1.1 (cyclo(K(N_3_)larllt). Our previous investigations of peptides L1.5 and cycloL1.1 revealed that both have high affinity for binding to domain I of the extracellular domain of EGFR, as well as enhanced stability in human serum [26]. 

The linear peptide L1.5 was synthesized using solid phase peptide synthesis (SPPS), as we have previously reported [26]. The cyclic peptide cycloL1.1 was synthesized in a similar fashion by SPPS, and then cyclized after removal from the resin. The peptides were purified by HPLC and analyzed by mass spectrometry and NMR. Analytical HPLC confirmed both peptides were >95% pure. 

**Scheme 2:**
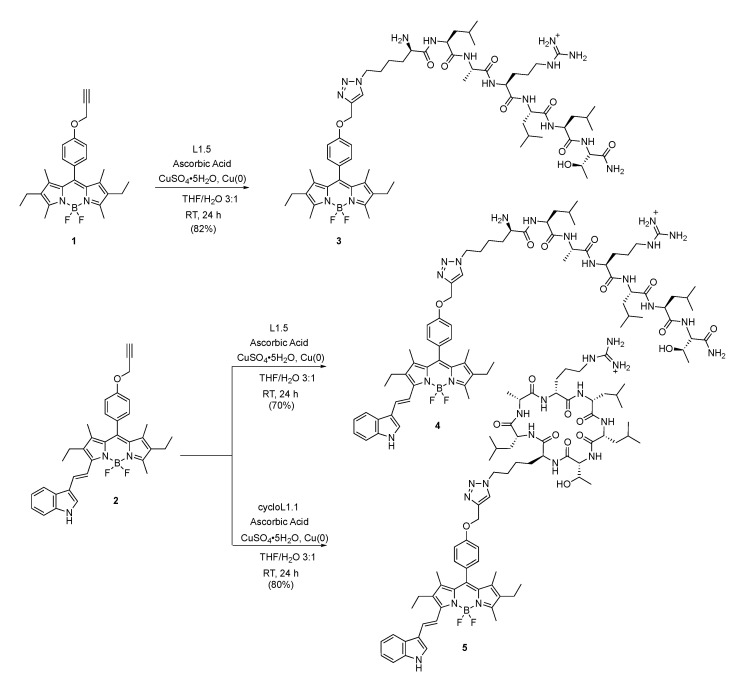
Click conjugation of azido peptides L1.5 and cycloL1.1 to BODIPYs **1** and **2**.

BODIPYs **1** and **2** were conjugated to peptides L1.5 or cycloL1.1 via click chemistry in the presence of CuSO_4_·5H_2_O, Cu(0), and l-ascorbic acid in THF/water [46], as shown in Scheme 2. To a solution of the BODIPY in THF/water 3:1 were added 1.5 equivalents of azido-peptide L1.5 dissolved in DMSO. Copper(0) in a catalytic amount was added to the resulting mixture followed by the addition of 1 equivalent of CuSO_4_·5H_2_O and l-ascorbic acid in distilled water. The catalytic amount of copper metal was added as a suitable reductant for the Cu(II) source, CuSO_4_·5H_2_O, in an aqueous solution to generate Cu(I), which is a potent stimulus for the formation of 1,2,3-triazoles. The reaction was allowed to stir for 24 h, monitored by MALDI-TOF mass spectrometry. Once the desired product was confirmed, the mixture was quenched with water. Initially, the resulting conjugate was purified through a molecular weight cut off (MWCO) tube of 1000 Daltons. This method was used to retain the conjugate in the tube, as the molecular weight for the desired product conjugates are all above this value, and any starting materials remaining in the mixture were removed. The resulting conjugates were dried and purified by RP-HPLC, eluted with a 1% TFA in H_2_O/0.1% TFA in CH_3_CN gradient (see Supporting Information) to afford the corresponding 1,2,3-triazole cycloadducts **3**, **4**, and **5** in excellent yields (Table 1). 

### 2.2. Spectroscopic Studies

Ultraviolet-Visible (UV/Vis) and fluorescence spectroscopy studies were employed to determine the spectroscopic properties of the BODIPYs and their conjugates. Experimental results from these studies are shown in Figure S19 (Supplementary Materials). BODIPY **1** was found to have excitation and emission maxima in the visible region of the electromagnetic spectrum at 525 and 540 nm, respectively. Extension of the π-conjugation system of the BODIPY through styryl functionalization via Knoevenagel condensation resulted in bathochromic shifts to 614 nm excitation and 648 nm emission maxima. As expected, the absorption and emission maxima did not change significantly after click conjugation with either the linear or cyclic peptides. Multiple shoulder peaks present in the absorption spectra of **4** and **5** reflect the possibility of electronic transitions between differing vibrational energy levels.

### 2.3. Surface Plasmon Resonance (SPR) Studies

We have previously explored the specificity of peptides L1.5 and cycloL1.1 for EGFR and determined that they both have a fast association with the EGFR protein’s extracellular domain through SPR studies [26]. To compare the binding affinity of the BODIPY-peptide conjugates **3**–**5** to EGFR in comparison to the peptide sequences alone, SPR [47,48] analyses were performed. All BODIPY-peptide conjugates and precursors were tested at concentrations up to 250 µM, and the results obtained are shown in Figures S10–S12 of the Supporting Information. Conjugates **3**, **4**, and **5** were diluted to desired concentrations with a solution of running buffer HBS-EP+ containing 8% of DMSO. 

For conjugate **5**, binding began at around 10 µM and further addition of conjugate did increase the sensorgram in a stepwise fashion, as seen in Figure S12 (Supplementary Materials). On the other hand, conjugate **3** binding changes in response began around 10 µM (Figure S10, Supplementary Materials) and there was a stepwise increase in the sensorgram with smooth association and dissociation with a total change of 450 RU, suggesting specific binding of the conjugate to the EGFR protein. At 250 µM, conjugate **4** showed a decrease in RU due to a saturation of binding (Figure S11, Supplementary Materials). On the other hand, conjugate **5** exhibited stepwise increase in binding from 25 to 250 µM concentrations (Figure S12, Supplementary Materials). Moreover, conjugate **5** sensorgram exhibited a smooth (and slow) increase in RU from 100 to 180 sec suggesting specific binding of conjugate to the EGFR extracellular domain, as shown with peptide cycloL1.1 [26]. Among the three conjugates studied, **3** and **5** exhibited relatively higher and specific binding to the EGFR protein. This is similar to what we observed for the free cyclic peptide which was characterized by a stepwise increase in binding with a slow and even increase in RU, rather than a rapid increase in the response units.

### 2.4. Cell Studies 

To assess the biological efficacy and behavior of the peptide-functionalized BODIPY conjugates, preliminary in vitro cellular studies, including dark cytotoxicity, phototoxicity and cellular uptake, were investigated against human squamous cell carcinoma HEp2 cell line, with high EGFR expression (~75%).

#### 2.4.1. Dark Toxicity

**Figure 2:**
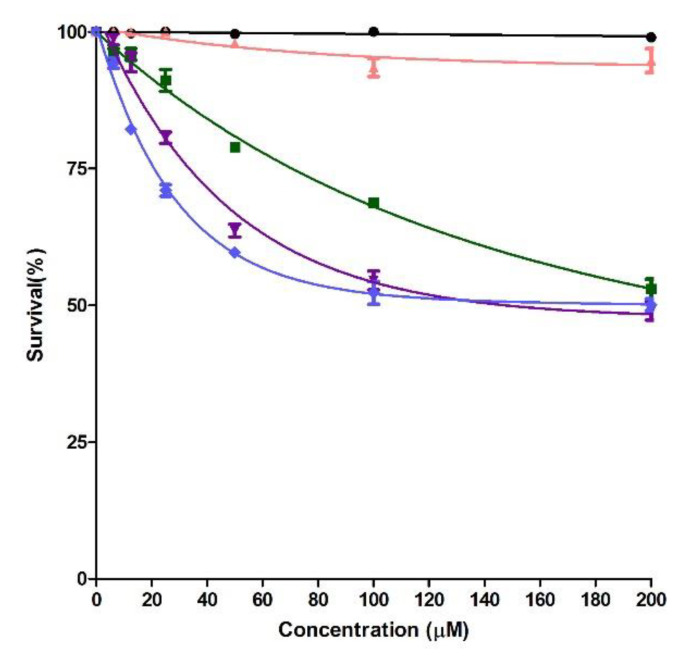
Dark toxicity results for BODIPYs **1** (black), **2** (green), **3** (pink), **4** (purple), and **5** (blue) in human carcinoma HEp2 cells.

The dark toxicity of all compounds was determined in HEp2 cells at concentrations up to 200 µM, using a CellTiter Blue (CTB) assay to determine cell viability. The results from these studies are shown in Figure 2. BODIPYs **1** and **2** (lacking peptide) were included in this study for comparison purposes. Interestingly, BODIPY **1** and conjugate **3**, both devoid of styryl and indole functionalities, were the least toxic compounds in this series. Conjugates **4** and **5** were found to be more cytotoxic than conjugate **3**, with calculated IC_50_ values of approximately 100 µM. On the other hand, BODIPY **2** bearing an indolyl styryl group was also found to be more toxic than **1**. These results suggest that the indolyl styryl moiety lends some cytotoxicity to compounds containing this functional group. This might be a result of the presence of the indole group, since it is a known pharmacophore and the basis of many drugs, such as indomethacin [44,49]. Conjugates **4** and **5** containing the linear and cyclic peptides, respectively, were found to have similar cytotoxicities, with the compound containing the cyclic peptide having slightly higher cytotoxicity than its linear counterpart at all concentrations investigated. This result might be due to the higher EGFR binding ability and uptake of the conjugate bearing the cyclic peptide.

#### 2.4.2. Phototoxicity

In addition to dark toxicity, phototoxicity was assessed for the BODIPYs and their conjugates, under a low light dose of ~1 J·cm^−2^ in human carcinoma HEp2 cells using the CTB assay to determine cell viability. Dose-dependent cell survival curves of each compound over the incubation period of 24 h are shown in Figure S20 (Supporting Information). The results obtained parallel those obtained for the dark cytotoxicity, with BODIPY **1** and its linear peptide conjugate **3** being the least toxic, whereas BODIPY **2** and its cyclic peptide conjugate **5** were found to be the most toxic, with IC_50_ values of approximately 100 µM. The phototoxicity of conjugate **3** is similar to that of its precursor BODIPY **1**, both of which were found to be the least cytotoxic in this series, suggesting that the conjugation of the linear peptide has little effect on the cytotoxicity of the BODIPY. On the other hand, BODIPY **2** and its conjugates **4** and **5** containing the indolyl styryl moiety, showed a significant increase in phototoxicity when compared to **1** and **3**, which lack the indolyl styryl group. Interestingly, conjugate **4** containing the linear EGFR-targeting peptide showed decrease in phototoxicity compared with its precursor **2**, although **4** showed increased cellular uptake in HEp2 cells than **2**. Nevertheless, all conjugates are considered to have low phototoxicity in HEp2 cells at the low light dose used in this study.

#### 2.4.3. Time-Dependent Cellular Uptake

**Figure 3:**
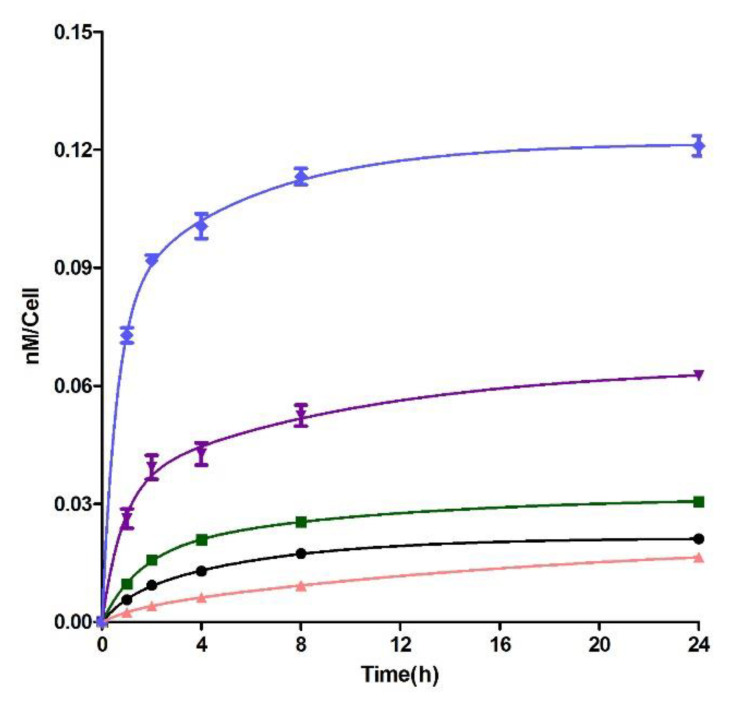
Cellular uptake results for BODIPYs **1** (black), **2** (green), **3** (pink), **4** (purple), and **5** (blue) in human carcinoma HEp2 cells.

Cellular uptake studies of all compounds were conducted in a time-dependent manner. Cellular uptake was determined in human carcinoma HEp2 cells over a 24 h time period at a non-toxic concentration of 10 µM for all compounds. A CyQuant^®^ cell proliferation assay was employed to determine cell numbers. The results from these studies are shown in Figure 3. BODIPYs **1** and **2** were also investigated to compare their cellular uptake with that of the peptide conjugates. After the addition of peptide-containing conjugates **4** and **5** to HEp2 cells, kinetics showed a marked uptake increase and rapid accumulation in cells in less than 4 h, after which a plateau in uptake is observed for the remaining of the 24 h time period. On the other hand, conjugate **3** and its parent BODIPY **1**, which are devoid of the indolyl styryl moiety, showed the lowest uptake in HEp2 cells. BODIPY **2** showed slightly higher uptake in HEp2 cells compared with **1** as a result of its indolyl styryl group. However, conjugates **4** and **5** containing the linear and cyclic peptide sequences, respectively, showed significant higher uptake compared with conjugate **3** in HEp2 cells, up to 7-fold increase in uptake. Both of these conjugates were readily taken up by HEp2 cells, with conjugate **5** showing the greatest uptake by far in this series. This result might be due to the higher affinity of the cyclic peptide for binding to EGFR. We previously reported that the d-amino acids in cyclo(K(N_3_)larllt) and its cyclic structure confer a more rigid conformation relative to the linear peptide that is more suitable for binding to domain I of EGFR [26]. In addition, the greater stability of the cyclic peptide might confer higher stability to conjugate **5** compared with **4**, thus allowing for a sustained higher uptake within cells over time. This observed enhanced cellular uptake for **5** may also contribute to its observed increase in photo- and dark-toxicity.

#### 2.4.4. Competitive Binding Studies 

**Figure 4:**
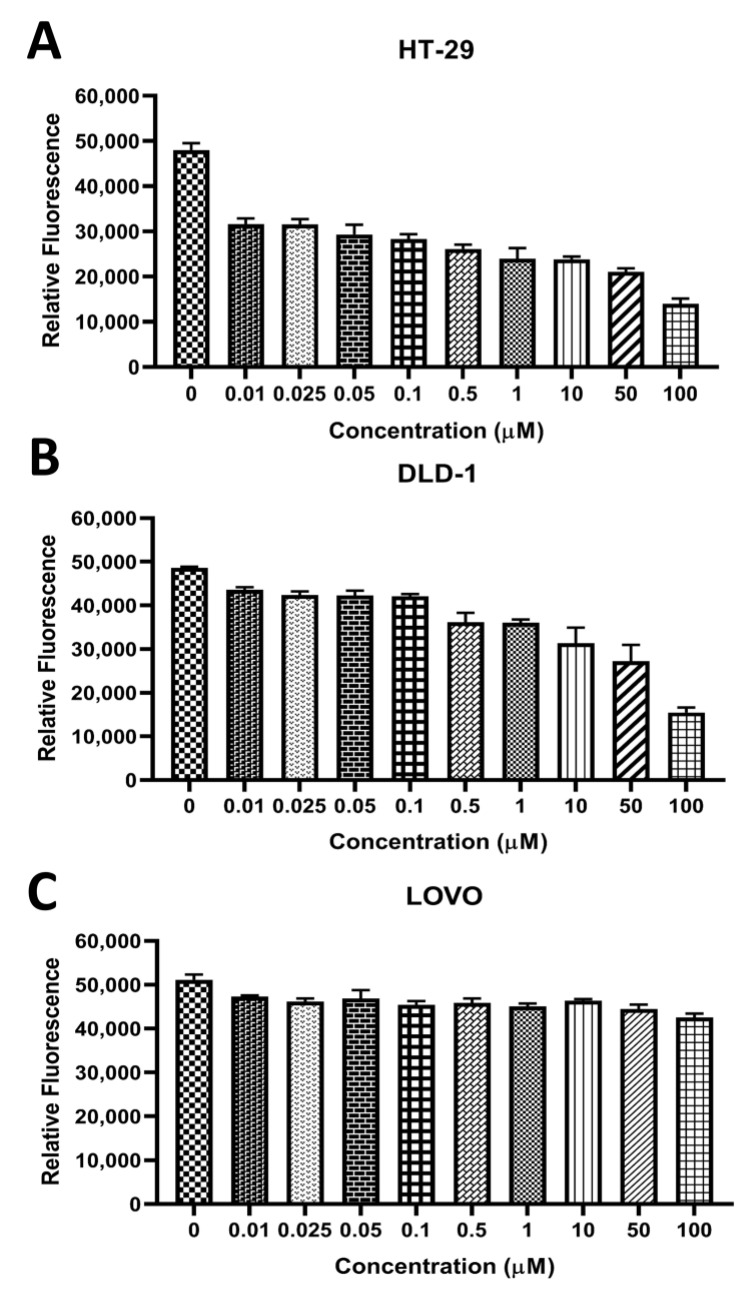
Evaluation of binding of conjugate **5** to EGFR on different cell lines that express differential amount of EGFR. Cyclic peptide cyclo(K(N_3_)larllt) (without BODIPY) was used with conjugate **5** and BODIPY fluorescence was monitored to observe the competitive binding. Binding of **5** to (**A**) HT-29 (high EGFR expression), (**B**) DLD-1 (moderate EGFR expression) and (**C**) LOVO (low EGFR expression). Zero concentration fluorescence refers to fluorescence without the addition of cyclo(K(N_3_)larllt). Concentrations on the *X*-axis are for various concentrations of cyclic peptide added with constant concentration of conjugate **5** (50 μM).

To evaluate the specificity of binding of the most promising conjugate **5** to EGFR overexpressing colon cancer cells, competitive binding experiments were carried out with cyclic peptide (cycloL1.1, without BODIPY) in the presence of conjugate **5**. In these studies, HT-29 (~78% EGFR expression), DLD-1 (~40% EGFR expression), and LOVO (~6% EGFR expression) were used [4]. When constant amount of conjugate **5** (50 μM) was added to HT-29 and DLD-1 cells in the presence of variable amount of cyclic peptide, a dose-dependent decrease in fluorescence intensity was observed as seen in Figure 4, suggesting that the cyclic peptide was able to replace conjugate **5** competitively, particularly in the high EGFR-expressing HT-29 cells. The highest inhibition of fluorescence intensity was observed in the HT-29 cells with the highest EGFR expression. When similar experiments were carried out in the presence of LOVO cells, there was no dose-dependent decrease in fluorescence intensity suggesting that both **5** and cyclic peptide bind only non-specifically to the LOVO cells. These results show that conjugate **5** binds specifically to EGFR over-expressing colon cancer cells.

## 3. Materials and Methods 

### 3.1. Synthesis

All reagents are commercially available and were purchased from Sigma-Aldrich (St. Louis, MO, USA) and VWR International (Radnor, PA, USA). All solvents and reagents used in the synthesis of conjugates were performed with reagents purchased from Sigma Aldrich peptide synthesis grade. Required amino acids were purchased from either Anaspec (Fremont, CA, USA), AAPPTec (Louisville, KY, USA) or Applied Biosystems (Foster City, CA, USA). Analytical thin-layer-chromatography (TLC) was performed on polyester backed TLC plates 254 (pre-coated, 200 µm, Sorbent Technologies, Norcross, GA, USA). Column chromatography was performed on silica gel (Sorbent Technologies, 60 Å, 40–63 µm). ^1^H-NMR and HSQC spectra were recorded using a Bruker AVIII-500 spectrometer (Birrica, MA, USA) (operating at 500 MHz for ^1^H-NMR and HSQC) in DMSO-d_6_. All chemical shifts are given in parts per millions (ppm) relative to tetramethylsilane (TMS, 0 ppm). All spectra were recorded at 308 K. High resolution mass spectra were obtained at the LSU Department of Chemistry Mass Spectrometry Facility using Bruker Omniflex MALDI Time-of-Flight Mass Spectrometer. The absorption measurements were carried out on a Varian 212 Cary 50 UV/Vis spectrophotometer (Palo Alto, CA, USA) and the steady-state fluorescence spectroscopic studies were performed on a PTI Quantum Master4/2006SE spectrofluorometer (Northampton, UK). All spectra were recorded at 298 K using non-degassed samples, spectroscopic grade solvents, and a 10-mm quartz cuvette. RP-HPLC was used to purify BODIPY-peptide conjugates, as shown in our previous work. The RP-HPLC system is comprised of a 2489 UV/Vis detector, a 2545 quaternary gradient module pump, and a FlexInject sample injector (Waters, Milford, MA, USA) to a purity of ≥95% (unless otherwise noted). To achieve full separation, X-Bridge BEH300 Prep C18 column (5 µm, 10 × 250 mm) armed with an X-Bridge BEH300 Prep guard column (300 Å, 5 µm, 10 × 10 mm) operating at a gradient of 50% A for 5 min, 50% A to 10% A over 1 min, 10% A to 0% A over 13 min, 0% A to 50% A over 2 min, then hold at 50% A for 5 min at a flow rate of 4 mL/min (unless otherwise noted). Conjugates UV detection was recorded for peptides at 220 nm, and for BODIPY at 540 and 640 nm. Fractions of HPLC purity (>95%) with the anticipated mass were combined and lyophilized. The chromatographs were analyzed using Empower 2 software. BODIPYs **1** and **2** were synthesized as previously described in the literature [42] (see Supplementary Materials). Peptides L1.5 and cycloL1.1 were synthesized as previously reported [26].

*General Procedure for 1,3-cycloaddition*: Alkynyl-functionalized BODIPY and azido-peptide (1.5 equiv.) were dissolved in a mixture of THF/H_2_O (1.8 mL, 3:1), followed by addition of Cu(0) (1.0 equiv.). A solution of CuSO_4_·5H_2_O (1 equiv.) and l-ascorbic acid (1 equiv.) in H_2_O was added to the reaction mixture. The solution was allowed to stir at room temperature for 24 h. Once the reaction was complete, the mixture was quenched with water and lyophilized. The fluffy product was then purified by RP-HPLC under varying gradients of water (with 0.1% TFA) and acetonitrile (with 0.1% TFA).

*Conjugate***3***(1,3,5,7-tetramethyl-2,6-diethyl-8-(4-phenoxymethyltriazole K(N_3_)LARLLT)-BODIPY):* This conjugate was obtained as a reddish orange solid (4.8 mg, 82%). MALDI-TOF *m*/*z* 1273.82; calc. for C_63_H_100_BF_2_N_16_O_9_^+^: 1273.79. For ^1^H-NMR and HSQC see Supporting Information.

*Conjugate***4***(1,3,7-trimethyl-5-indolylstyryl-2,6-diethyl-8-(4-phenoxymethyltriazole K(N_3_)LARLLT)-BODIPY)*: This conjugate was obtained as a dark blue solid (3.49 mg, 70%). MALDI-TOF *m*/*z* 1400.87; calc. for C_73_H_106_BF_2_N_16_O_9_^+^: 1400.89. For ^1^H-NMR and HSQC see Supporting Information.

*Conjugate***5***(1,3,7-trimethyl-5-indolylstyryl-2,6-diethyl-8-(4-phenoxymethyltriazole cycloK(N_3_)larllt)-BODIPY)*: This conjugate was obtained as a deep blue solid (6.1 mg, 80%). MALDI-TOF *m*/*z* 1383.91; calc. for C_72_H_102_BF_2_N_16_O_9_^+^: 1383.81. For ^1^H-NMR and HSQC see Supporting Information.

### 3.2. Spectroscopy Studies

UV-Visible and emission spectroscopy studies were measured using a Varian Cary spectrophotometer (Palo Alto, CA, USA) and a Perkin Elmer LS55 spectrophotometer (Waltham, MA, USA) at room temperature. Quartz cuvettes (1 cm path length) were used for each study. To analyze the data, integrated absorbance against the corresponding solution concentrations were plotted to conclude the extinction coefficients (ε) at the maximum absorption.

### 3.3. Surface Plasmon Resonance (SPR) Studies

Surface plasmon resonance analysis was conducted using the extracellular domain of EGFR protein (Leinco Technologies, St. Louis, MO, USA) immobilized on a CM5 sensor chip via standard amine coupling with a Biacore X100 (GE Healthcare Life Sciences, Marlborough, MA, USA) as described in our previous publications [22,26]. The running buffer used was 8% DMSO in 1X HBS-EP+ (0.01 M HEPES, 0.15 M NaCl, 3 mM EDTA, 0.005% Tween, pH 7.5) (GE Healthcare Biosciences, Chicago, IL, USA), at a flow rate of 5 µL/min. DMSO was added to the running buffer and peptide solutions to enhance the solubility of the peptides studied. The solutions were prepared in running buffer and filtered using a 0.45 µm filter. All SPR sensorgrams relay the rates of association and dissociation of analytes at concentrations of 0 to 250 µM performed at room temperature.

### 3.4. Cell Studies

For cellular studies, commercially available reagents and culture media were purchased from Life Technologies (Carlsbad, CA, USA) and human carcinoma HEp2 (ATCC CCL-23) cells were purchased from the American Type Culture Collection (ATCC) (Manassas, Virginia, USA). HEp2 cells were cultured and maintained at 37 °C under 5% CO_2_ and 95% humidity in minimum essential media (MEM) supplemented with 10% fetal bovine serum (FBS) and 1% penicillin-streptomycin (P/S).

#### 3.4.1. Dark Cytotoxicity

Stock solutions (32 mM) for compounds **1**, **2**, **3**, **4,** and **5** were prepared using 100% DMSO as the solvent. Working solutions were then prepared at concentrations of 0, 6.25, 12.5, 25, 50, 100, and 200 µM by diluting stock solutions with culture medium. HEp2 cells were exposed to the working solutions up to 200 µM with 24 h incubation (5% CO_2_, 95% humidity, 37 °C). After 24 h treatment, the loading medium was removed, and cells were washed thrive with phosphate buffered saline (PBS) solution to remove residual compound. Then, medium containing 20% CellTiter Blue (CTB) (Promega, Madison, WI, USA) was added to the cells and allowed to incubate it for another 4 h. Following the incubation period, cell viability was assessed by determining fluorescence intensity at 570/615 nm using a BMG FLUOstar Optima microplate reader (BMG Labtech, Cary, NC, USA). The fluorescence intensity was normalized to 100% for untreated cells and the results are expressed as a percentage of viable cells.

#### 3.4.2. Phototoxicity

HEp2 cells were exposed to working solutions of each compound up to 100 µM (0, 3.125, 6.25, 12.5, 25, 50, and 100 µM) and incubated for 24 h (5% CO_2_, 95% humidity, 37 °C). The loading medium was removed and the cells were washed with PBS solution. Fresh culture medium was introduced to the cells, followed by light exposure using a halogen lamp (600 W) light source that included a beam turning mirror (200 nm to 30 µm spectral range, Newport) and a water filter (transmits radiation 250–950 nm), for 20 min. The cells received a total light dose of approximately 1 J·cm^−2^. After 20 min light exposure, the cells were returned to the incubator for another 24 h. The culture medium was replaced with media containing 20% CellTiter Blue and the cells incubated for 4 h. Cell viability was assessed as described above.

#### 3.4.3. Time-Dependent Cellular Uptake

Cellular uptake was investigated in HEp2 cells exposed to 10 µM solutions of compounds **1**, **2**, **3**, **4,** and **5** at incremental time intervals (0, 1, 2, 4, 8, and 24 h). At the end of the allotted time, the loading medium was removed, and the cells washed with PBS solution to remove residual compound not taken up by the cells. Cells were solubilized using 0.25% Triton X-100 in PBS. Compound standard curves were created by diluting the 32 mM stock solutions with 0.25% Triton X-100 in PBS to final concentrations of 10, 5, 2.5, 1.25, 0.625, and 0.3125 µM. A cell standard curve was created by plating 10,000, 20,000, 40,000, 60,000, 80,000, and 100,000 cells per well (five replicates for each concentration). To quantify cells, CyQuant Cell Proliferation Assay (Invitrogen, Carlsbad, CA, USA) was used. The concentration of the compounds was determined using the standard curves and a BMG FLUOstar Optima microplate reader at 485/520 nm. Cellular uptake results are expressed in nM of compound per cell.

#### 3.4.4. Competitive Binding Studies

Cells HT-29 (~78% EGFR) DLD-1 (~37% EGFR) and LOVO (~6% EGFR) with high, moderate, and low EGFR expressions, respectively, were used in these studies. About ten thousand cells of each cell line were coated in a 96 well clear bottom tissue culture black plate and incubated overnight at 37 °C with 5% CO_2_. After 24 h, the medium was removed, and different concentrations (0.25 to 100 μM) of cyclo(K(N_3_)larllt) with a constant concentration (50 μM) of conjugate **5**, was added to the wells in triplicate. PBS was used for dilution of compounds. The plate was then covered in aluminum foil and incubated at 37 °C with 5% CO_2_. After 45 min, each well was washed twice with 100 μL of PBS, and fluorescence was determined with a microplate reader using an excitation wavelength of 614 nm and an emission wavelength of 648 nm. Autofluorescence values were subtracted from each well. A plot of concentrations vs. relative fluorescence was plotted. Results in Figure 4 are expressed as ±S.E.

## 4. Conclusions

Two terminal alkyne-functionalized BODIPY dyes and two EGFR azido-peptide ligands, one linear and one cyclic, were conjugated via click chemistry in 70–82% yields. Of all the conjugates, **4** and **5** bearing an indolyl styryl group showed increased cellular uptake and cytotoxicity compared with **3**, which bears no indolyl styryl group. However, the presence of the cyclic peptide in conjugate **5** induced the highest accumulation in HEp2 cells that overexpress EGFR of all compounds in this series. This might be a result of the more rigid conformation of the cyclic peptide, which is more suitable for binding to EGFR, compared with the linear peptide. SPR results suggest that all conjugates bind to EGFR, in particular, conjugate **5**. Competitive binding studies using three cell lines with different EGFR expression showed that conjugate 5 specifically binds to colon cancer cells overexpressing EGFR (HT-29), while non-specific binding was also observed in cells with low EGFR expression (LOVO). The click conjugation of fluorophores to azide-functionalized peptides is anticipated to provide a resourceful methodology for the preparation of vast arrays of functionalized BODIPYs possessing potential utility in in vivo imaging.

## Figures and Tables

**Table 1:** Molecular weights and click reaction yields of BODIPY-peptide conjugates.

Conjugate	Molecular Weight (g/mol)	Peptide	%Yield
**3**	1273.79	L1.5	82%
**4**	1400.89	L1.5	70%
**5**	1383.81	cycloL1.1	80%

## Data Availability

The data presented in this study are available in Supplementary Materials.

## Supplementary Materials

The following are available online, Figure S1: HPLC for 3, Figure S2: HPLC for 4, Figure S3: HPLC for 5, Figure S4: MALDI-TOF for 3, Figure S5: MALDI-TOF-TOF for 3, Figure S6: MALDI-TOF for 4, Figure S7: MALDI-TOF-TOF for 4, Figure S8: MALDI-TOF for 5, Figure S9: MALDI-TOF-TOF for 5, Figure S10: SPR sensorgram for 3, Figure S11: SPR sensorgram for 4, Figure S12: SPR sensorgram for 5, Figure S13: ^1^H-NMR spectrum for 3, Figure S14: HSQC NMR spectrum for 3, Figure S15: ^1^H-NMR spectrum for 4, Figure S16: HSQC NMR spectrum for 4, Figure S17: ^1^H-NMR spectrum for 5, Figure S18: HSQC NMR spectrum for 5, Figure S19: Normalized UV-Vis and fluorescence spectra, Figure S20: Phototoxicity results in human HEp2 cells.
