# Supplementary material for: Click Conjugation of Boron Dipyrromethene (BODIPY) Fluorophores to EGFR-Targeting Linear and Cyclic Peptides
Source: Molecules. 2021 Jan 23;26(3):593. doi: 10.3390/molecules26030593 (PMC7865655; doi:10.3390/molecules26030593)
Supplement: Supplementary file 1 [file molecules-26-00593-s001.pdf]

Supporting Information

Click Conjugation of Boron Dipyrromethene (BODIPY) Fluorophores to EGFR-targeting Linear and Cyclic Peptides

Tyrslai M. Williams[1], Nichole E. M. Kaufman[1], Zehua Zhou[1], Sitanshu S. Singh[2], Seetharama D. Jois[2], and M. Graça H. Vicente[1,*]

Table of Contents

Experimental procedure for BODIPYs **1** and **2**…………………………..……………S2

Figure S1. HPLC for **3**…………………………………………………………...…S3

Figure S2. HPLC for **4**……………………………………………………………...S3

Figure S3. HPLC for **5**……………………………………………………………...S4

Figure S4. MALDI-TOF of **3**…………………………………………...……......S4

Figure S5. MALDI-TOF-TOF of **3**…………………………………….……...S5

Figure S6. MALDI-TOF of **4**………………………………………….......…...S5

Figure S7. MALDI-TOF-TOF of **4**………………………………………….…...S6

Figure S8. MALDI-TOF of **5**……………………………………………….……...S6

Figure S9. MALDI-TOF-TOF of **5**……………………………………….………...S7

Figure S10. SPR sensorgram of **3**……………………………...……………...S8

Figure S11. SPR sensorgram of **4**…………………………………...………...S8

Figure S12. SPR sensorgram of **5**……………………………………...……...S9

Figure S13. ^{1}H NMR spectrum of **3**…………………………………….….……...S11

Figure S14. HSQC NMR spectrum for **3**……………………………….….……...S12

Figure S15. ^{1}H NMR spectrum for **4**…………………………………………...S13

Figure S16. HSQC NMR spectrum for **4**……………………………….….……...S14

Figure S17. ^{1}H NMR spectrum for **5**……………………………………………...S15
Figure S18. HSQC NMR spectrum for **5**…………………………………….….…...S16
Figure S19. Normalized UV-Vis and Fluorescence Spectra…………………...…..S17
Figure S20. Phototoxicity results in human HEp2 cells…….…………………….…..S17

Experimental procedure for BODIPYs 1 and 2:

4-propargyloxybenzaldehyde: 4-hydroxybenzaldehyde (2.00 g, 16.38 mmol) and propargyl bromide (7.79 g, 65.51 mmol,) were added to a solution of potassium carbonate (13.58 g, 98.3 mmol) in 100 mL acetone. The reaction mixture was heated to reflux overnight and then cooled to room temperature. The heterogeneous solution was filtered off to remove solid potassium carbonate and the solvent was removed under vacuum. The crude product was dissolved in dichloromethane (20 mL) and washed successively with water twice (20 mL), 5% $NaHCO_3$ (20 mL) and brine (20 mL) once each. The organic extracts were combined, dried over Na_2SO_4 and concentrated under vacuum. The resulting crude product was purified by column chromatography using pure dichloromethane as the eluent to afford 2.28 g of the tile compound as pale-yellow solid (quantitative). Spectroscopic data are in agreement with literature.

BODIPY 1: 3-ethyl-2,4-dimethyl pyrrole (3.51 g, 28.47 mmol) and 4-propargylbenzaldehyde (0.6490 g, 4.0584 mmol) were dissolved in dry dichloromethane (281 mL) under an inert gas atmosphere. Two drops of BF_3•OEt_2 were added and the reaction mixture was stirred overnight at room temperature. When the aldehyde was consumed (monitored by TLC), a solution of DDQ (3.88 g, 17.08 mmol) in dry dichloromethane (35 mL) was added via syringe at 0°C. After stirring for 20 min at room temperature, triethylamine (13.89 mL) was added dropwise and the reaction mixture was stirred for another 30 min at room temperature. Further, BF_3•Et_2O (19.32 mL) was added dropwise at 0°C and the mixture was stirred for 10 h at room temperature. Once TLC indicated reaction completion, the reaction mixture was passed through a short plug of silica gel to separate from any oxidized dipyrromethene derivatives and starting materials. The solvent was removed under reduced pressure and the residue was dissolved in dichloromethane (100 mL) and stirred overnight at room temperature. The subsequent reaction mixture was extracted with 0.1 M HCl (2 x 100 mL) to remove excess DDQ, and with brine (1 x 100 mL). The organic extracts were collected, dried over Na_2SO_4 and the solvent was evaporated. The crude product was purified by column chromatography using hexane/dichloromethane (1/1, v/v) as eluent. The orange-green fluorescent fraction containing the desired

BODIPY **1** was collected and recrystallized from dichloromethane/hexane mixtures to yield the title compound as a lustrous orange-red solid. Spectroscopic data are in agreement with literature.

BODPY 2: BODIPY **1** (0.0122 g, 0.02809 mmol) and indole-3-carbaldehyde (0.01223 g, .08427 mmol) were added to a 150 mL triple necked round bottom flask containing a mixture of dry toluene (20 ml), piperidine (0.032 ml) and glacial acetic acid (0.019 ml). The mixture was refluxed overnight. After completion of the reaction (monitored via TLC), the mixture was cooled to room temperature and the solvent was removed under vacuum. The residue was purified by silica gel column chromatography using dichloromethane/hexane (2/1, v/v) as eluent. The first fraction with red fluorescence was dried under vacuum, affording BODIPY **2** as a blue solid. Spectroscopic data are in agreement with literature.

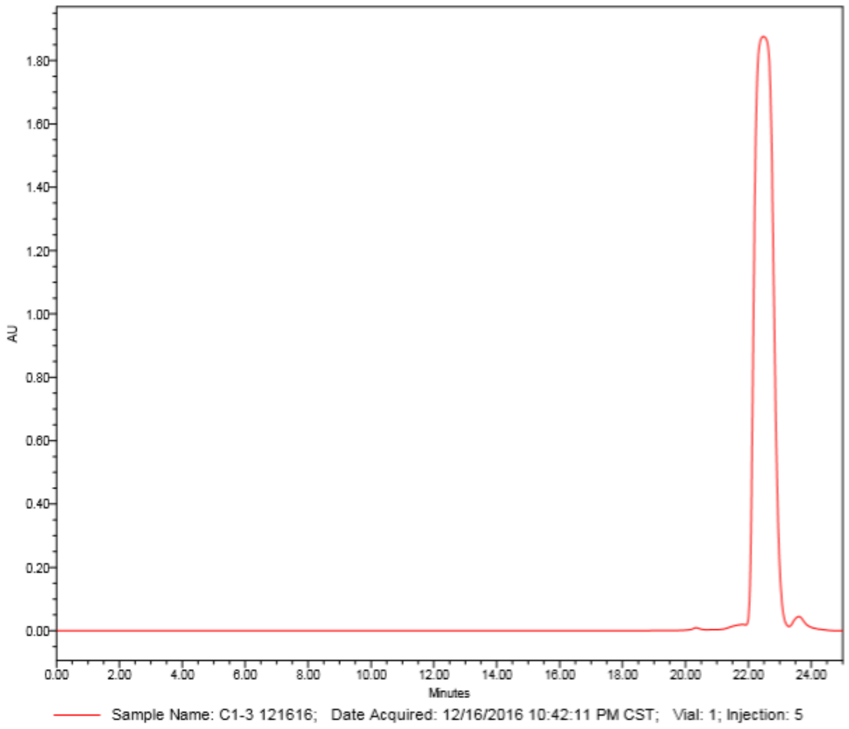

Figure S1. HPLC for conjugate **3**; r.t. 22.498 min, % Area = 95.04.

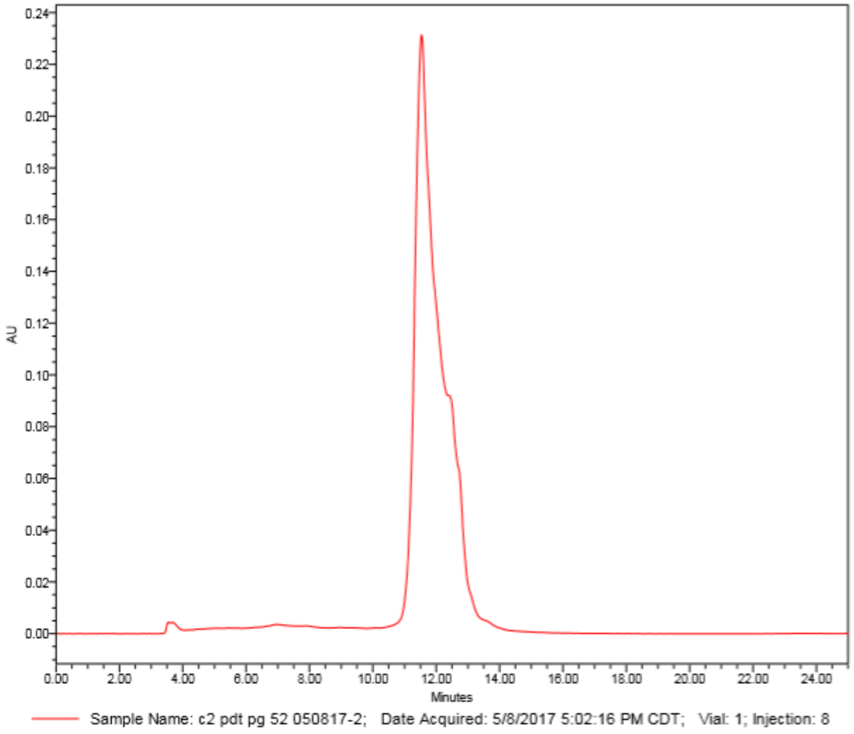

Figure S2. HPLC for conjugate **4**; r.t. 11.525 min, % Area = 98.06.

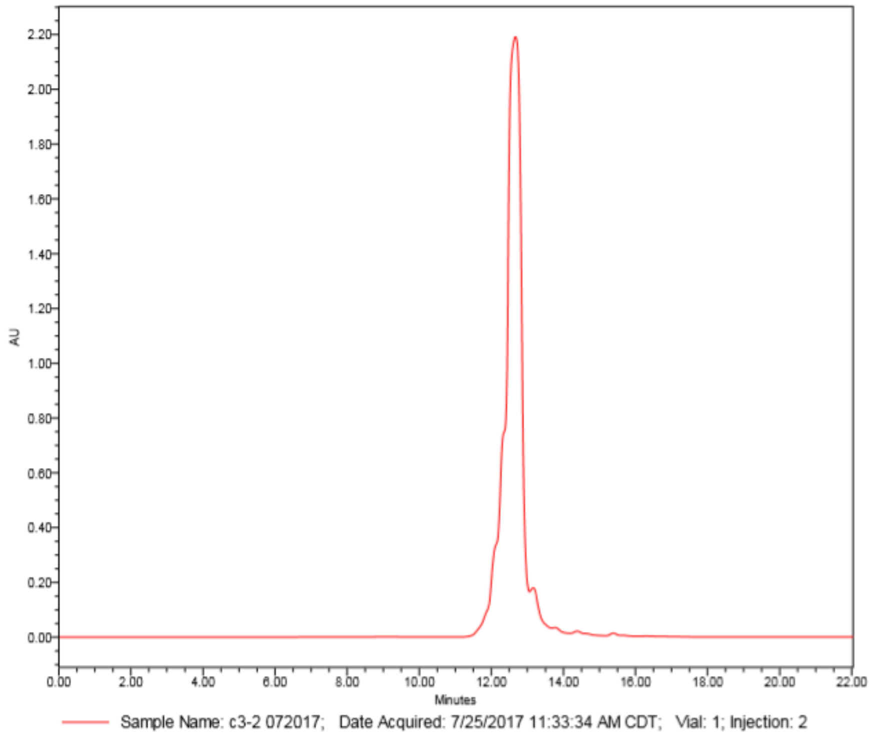

Figure S3. HPLC for conjugate **5**; r.t. 12.667 min, % Area = 95.21.

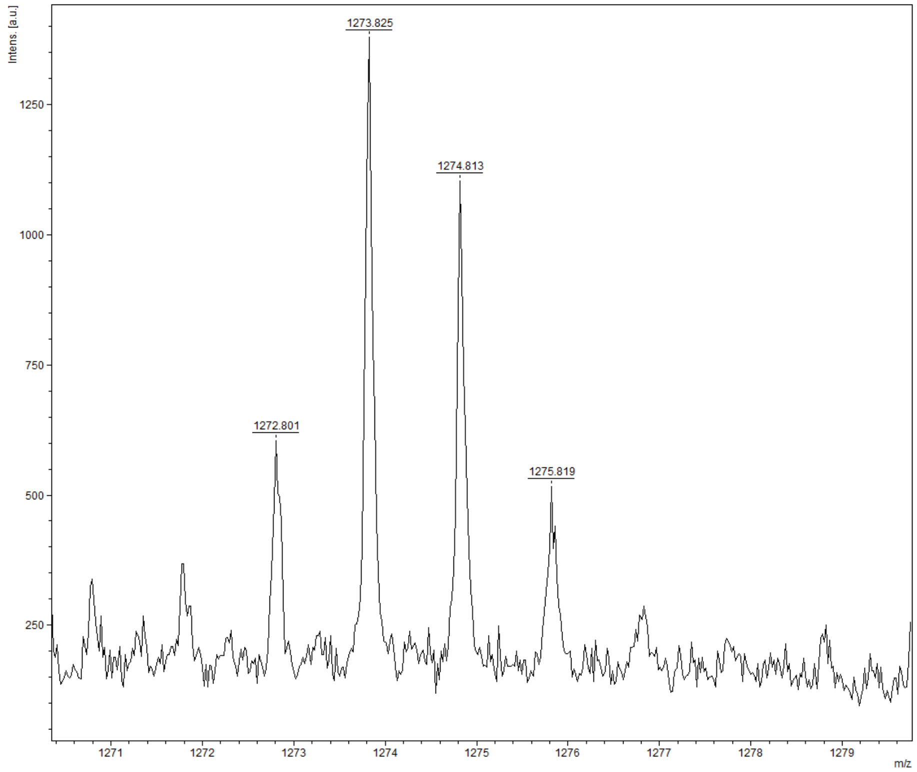

Figure S4. MALDI-TOF of conjugate **3**.

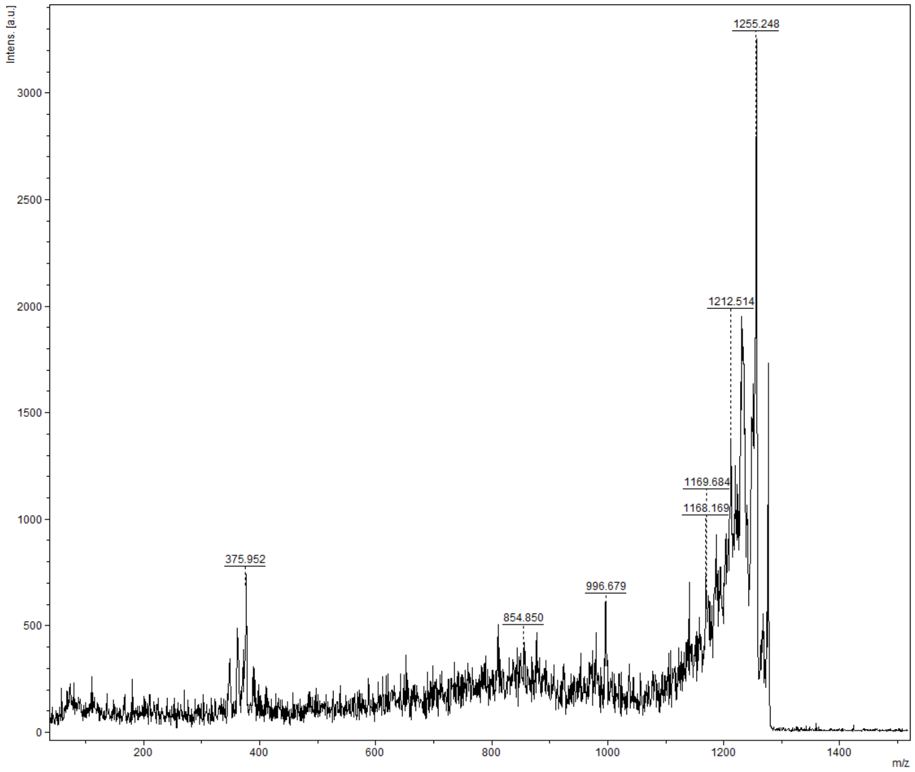

Figure S5. MALDI-TOF-TOF of conjugate **3**.

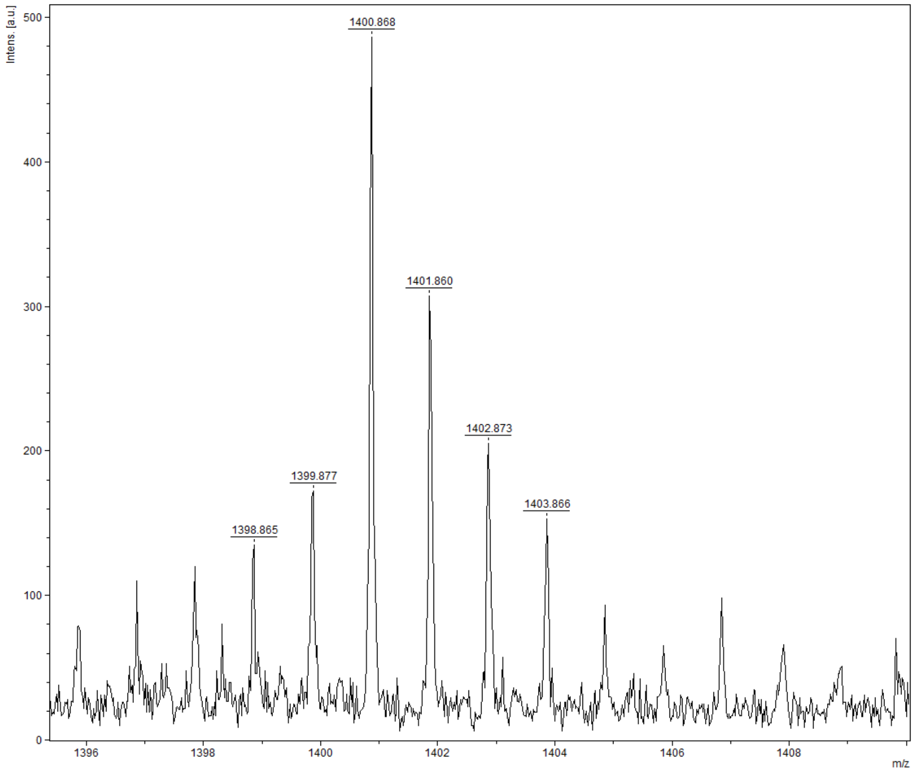

Figure S6. MALDI-TOF of conjugate **4**.

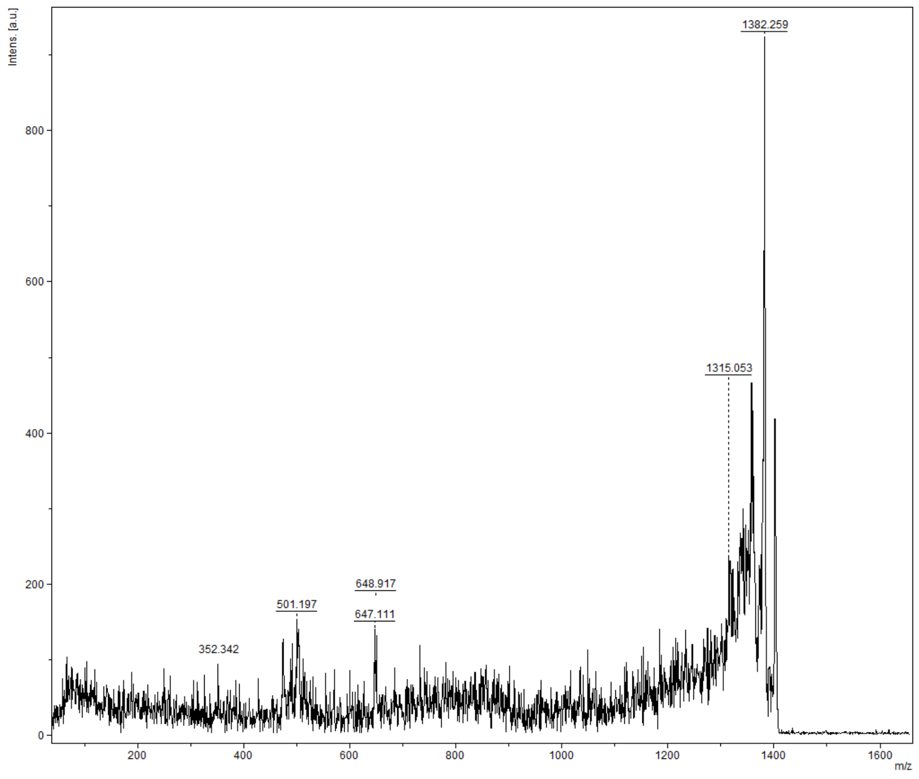

Figure S7. MALDI-TOF-TOF of conjugate **4**.

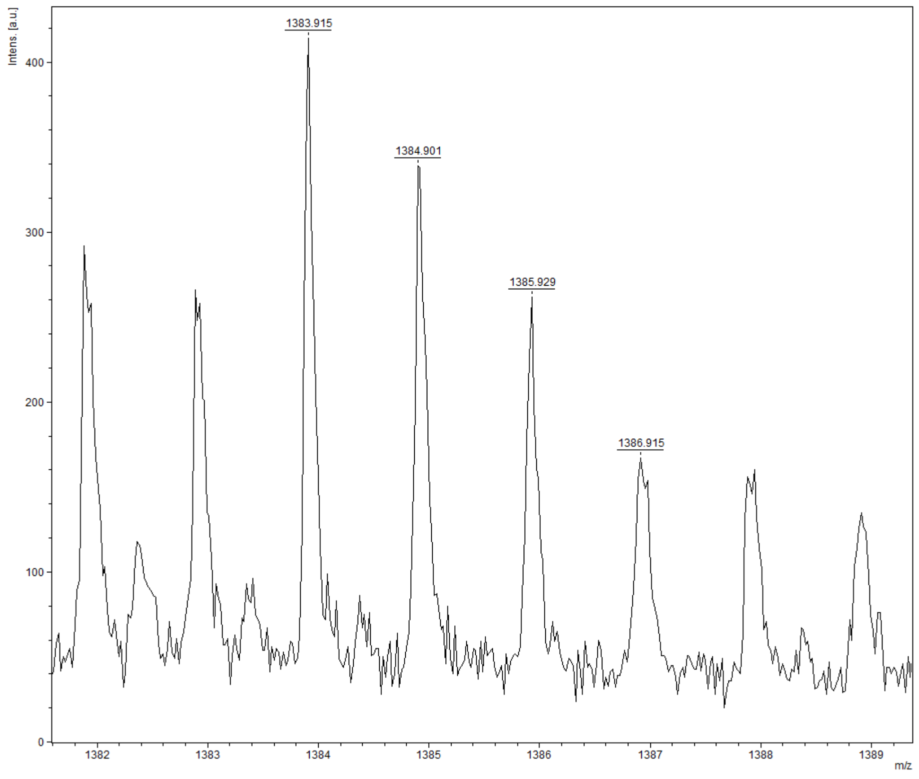

Figure S8. MALDI-TOF of conjugate **5**.

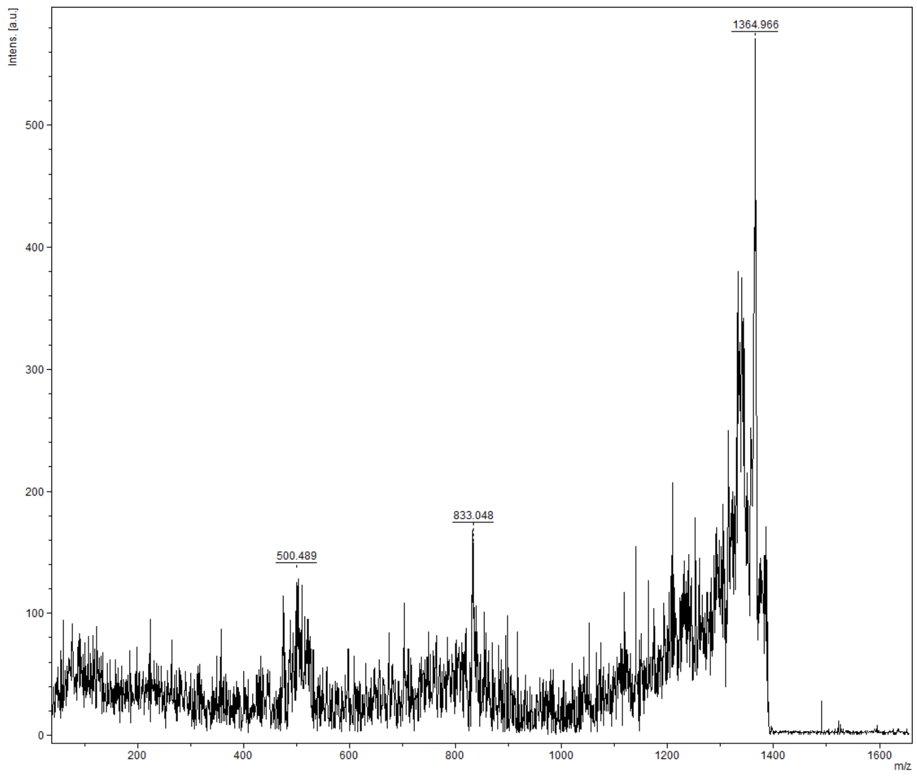

Figure S9. MALDI-TOF-TOF of conjugate **5**.

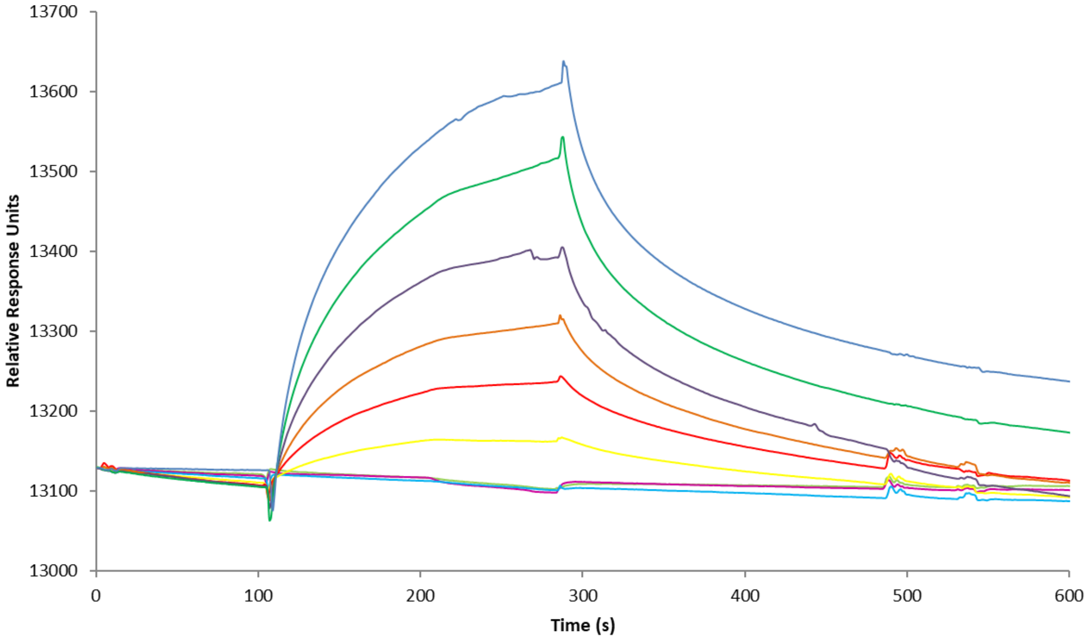

Figure S10. SPR sensorgram for conjugate **3** in 8% DMSO. [*Concentrations: 0μM (lime green); 0.5μM (fushia); 1μM (cyan); 10μM (yellow); 25μM (red); 50μM (orange); 100μM (purple); 200μM (green); 250μM (blue)*].

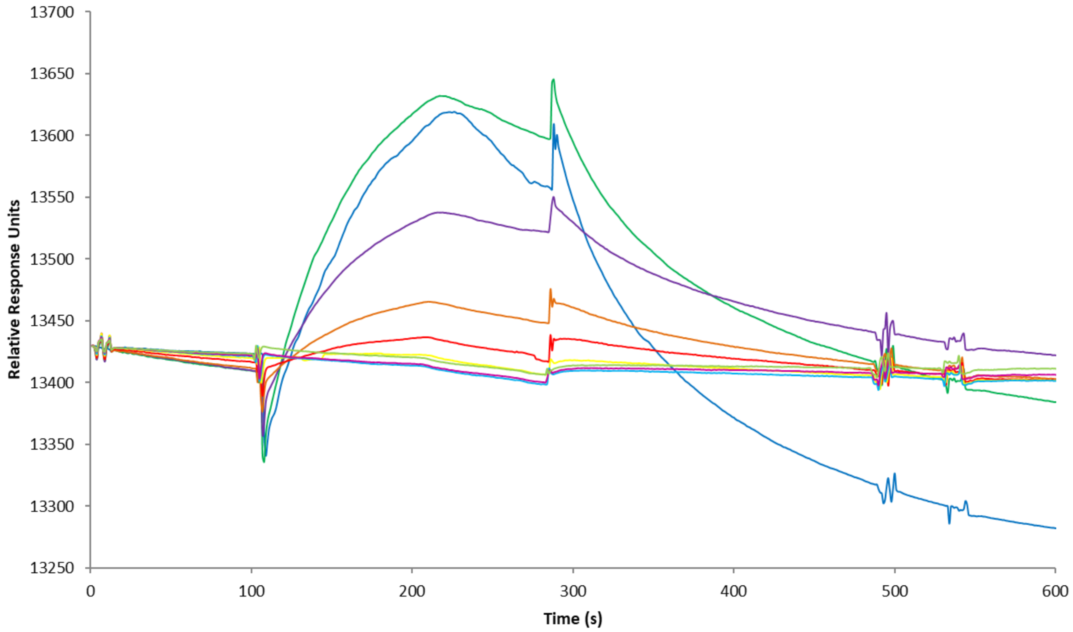

Figure S11. SPR sensorgram for conjugate **4** in 8% DMSO. [*Concentrations: 0μM (lime green); 0.5μM (fushia); 1μM (cyan); 10μM (yellow); 25μM (red); 50μM (orange); 100μM (purple); 200μM (green); 250μM (blue)*].

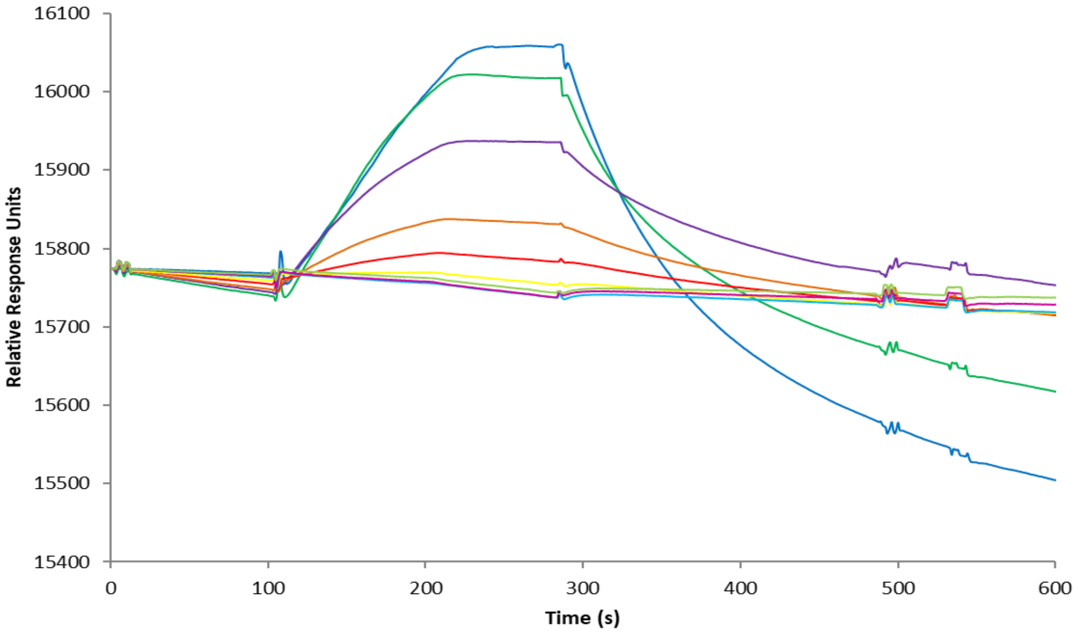

Figure S12. SPR sensorgram for conjugate **5** in 8% DMSO. [*Concentrations: 0μM (lime green); 0.5μM (fushia); 1μM (cyan); 10μM (yellow); 25μM (red); 50μM (orange); 100μM (purple); 200μM (green); 250μM (blue)*].

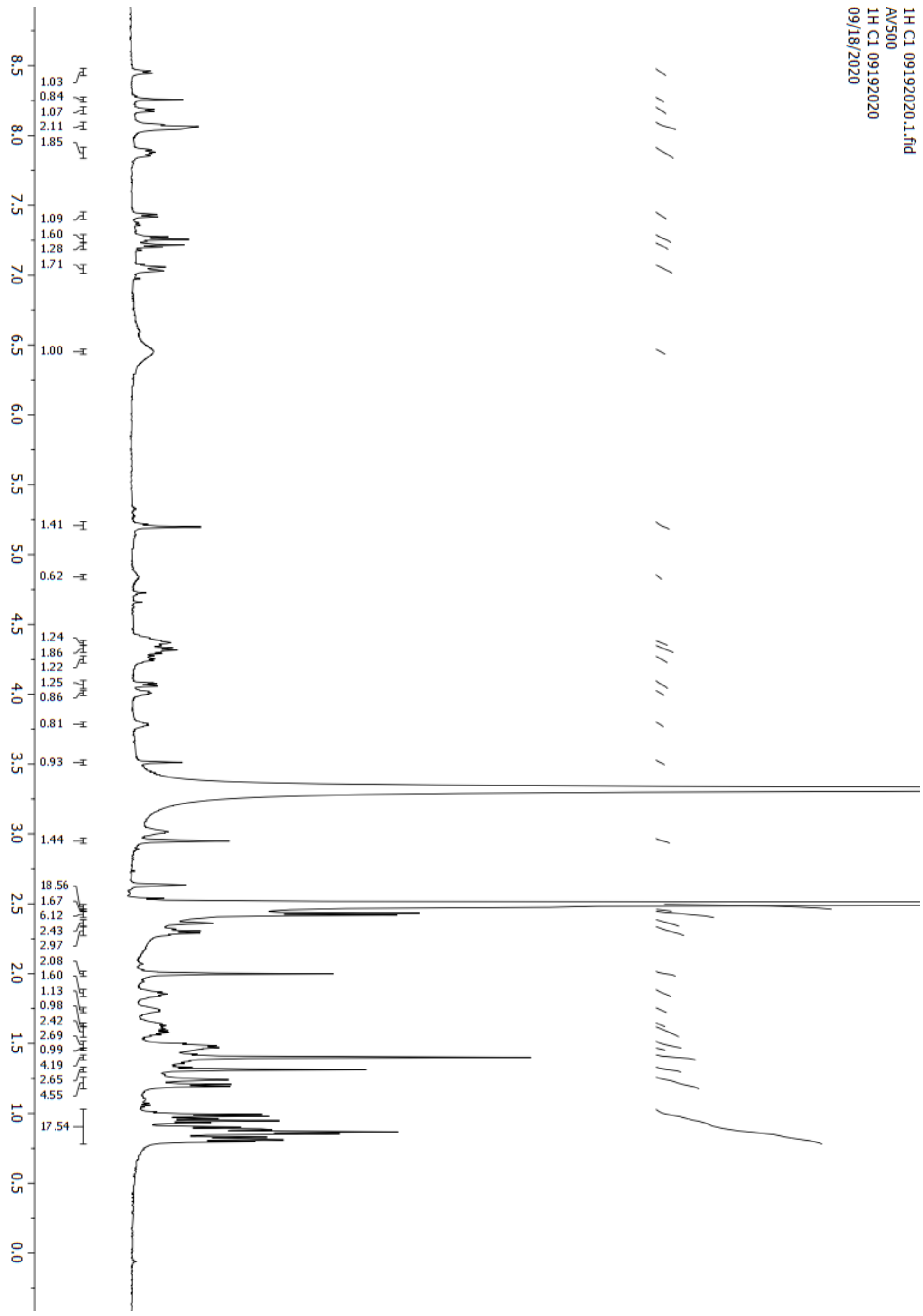

Figure S13. ^{1}H NMR of conjugate **3**.

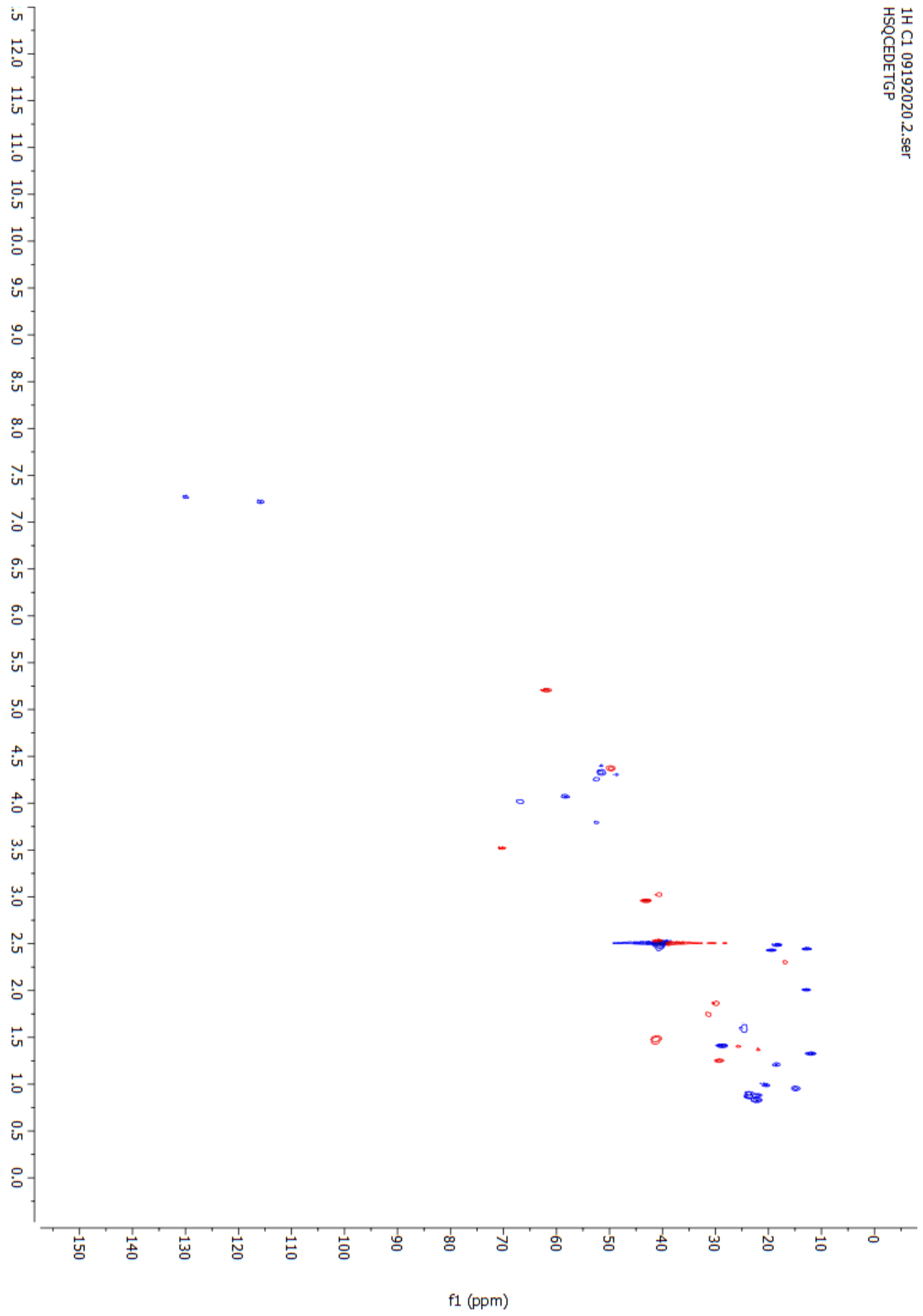

Figure S14. HSQC NMR of conjugate **3**.

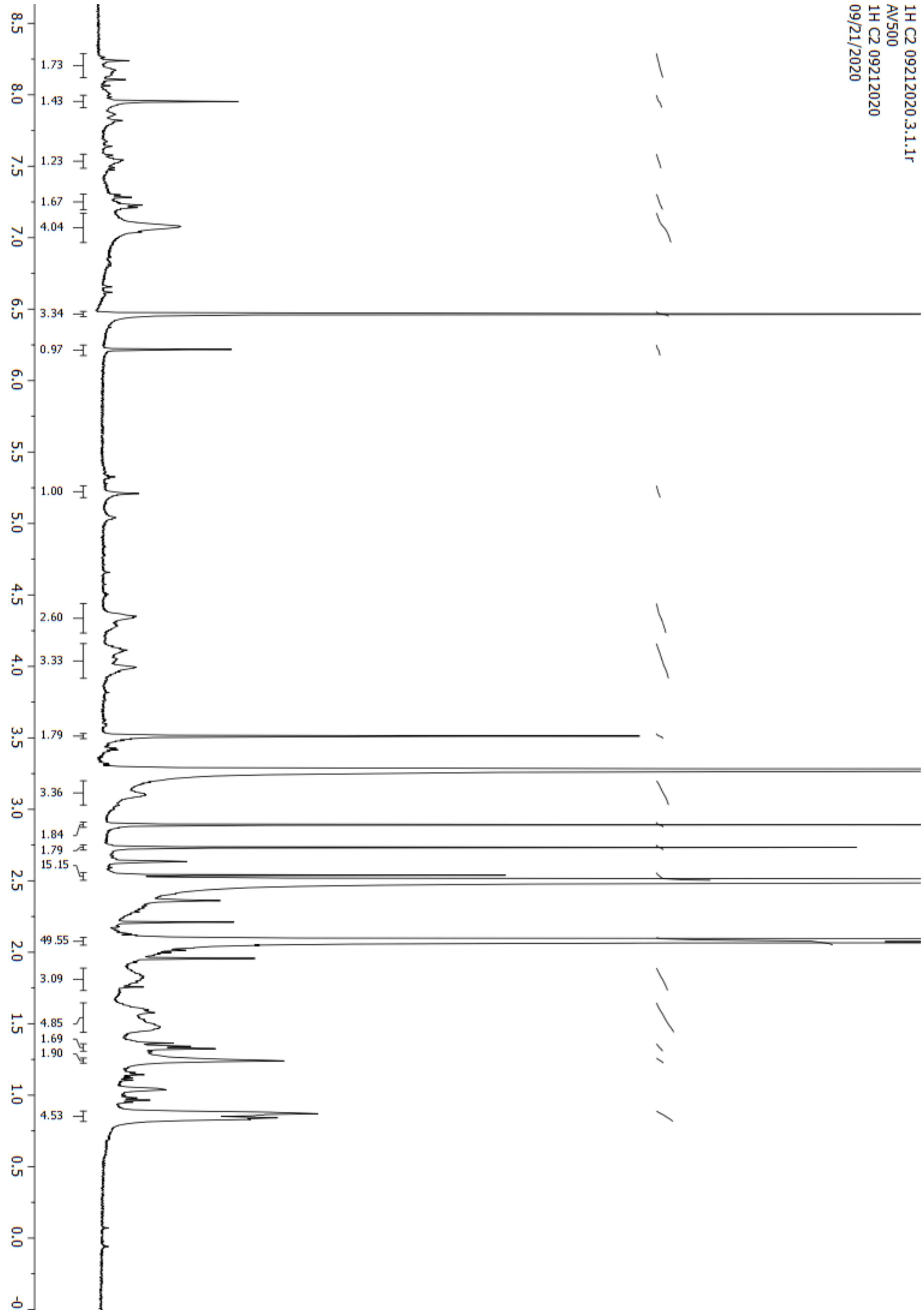

Figure S15. ^{1}H NMR of conjugate **4**.

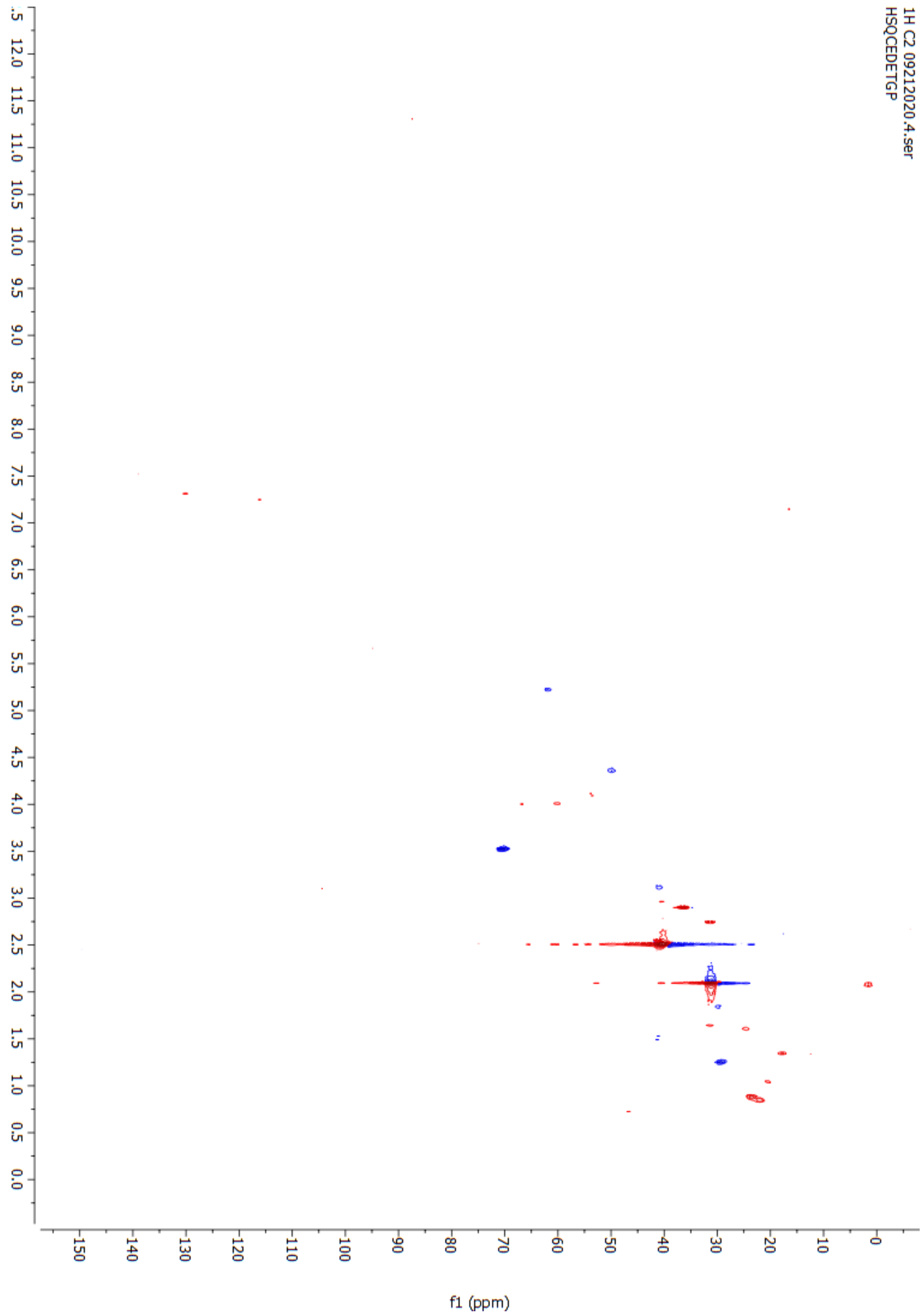

Figure S16. HSQC NMR of conjugate **4**.

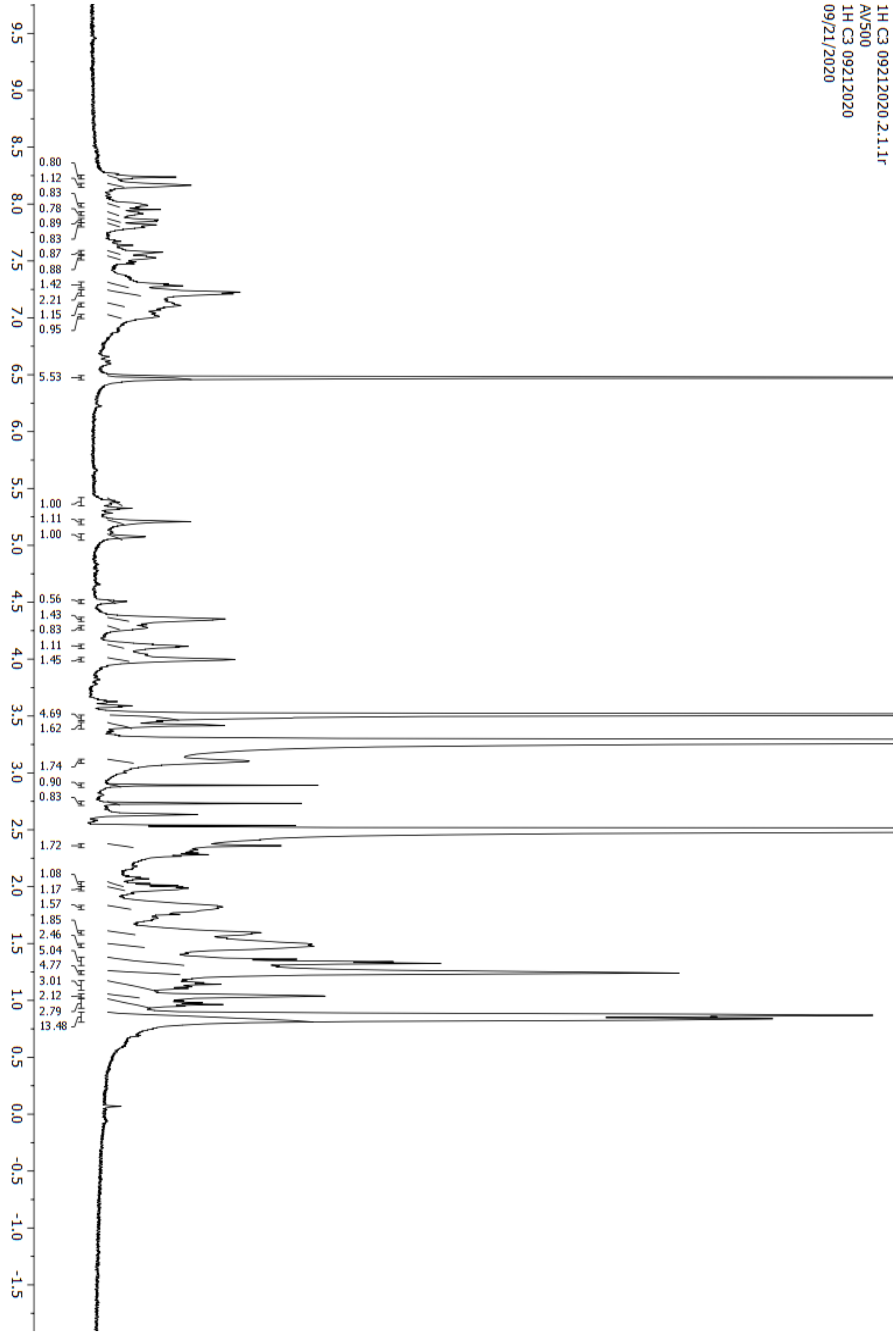

Figure S17. ^{1}H NMR of conjugate **5**.

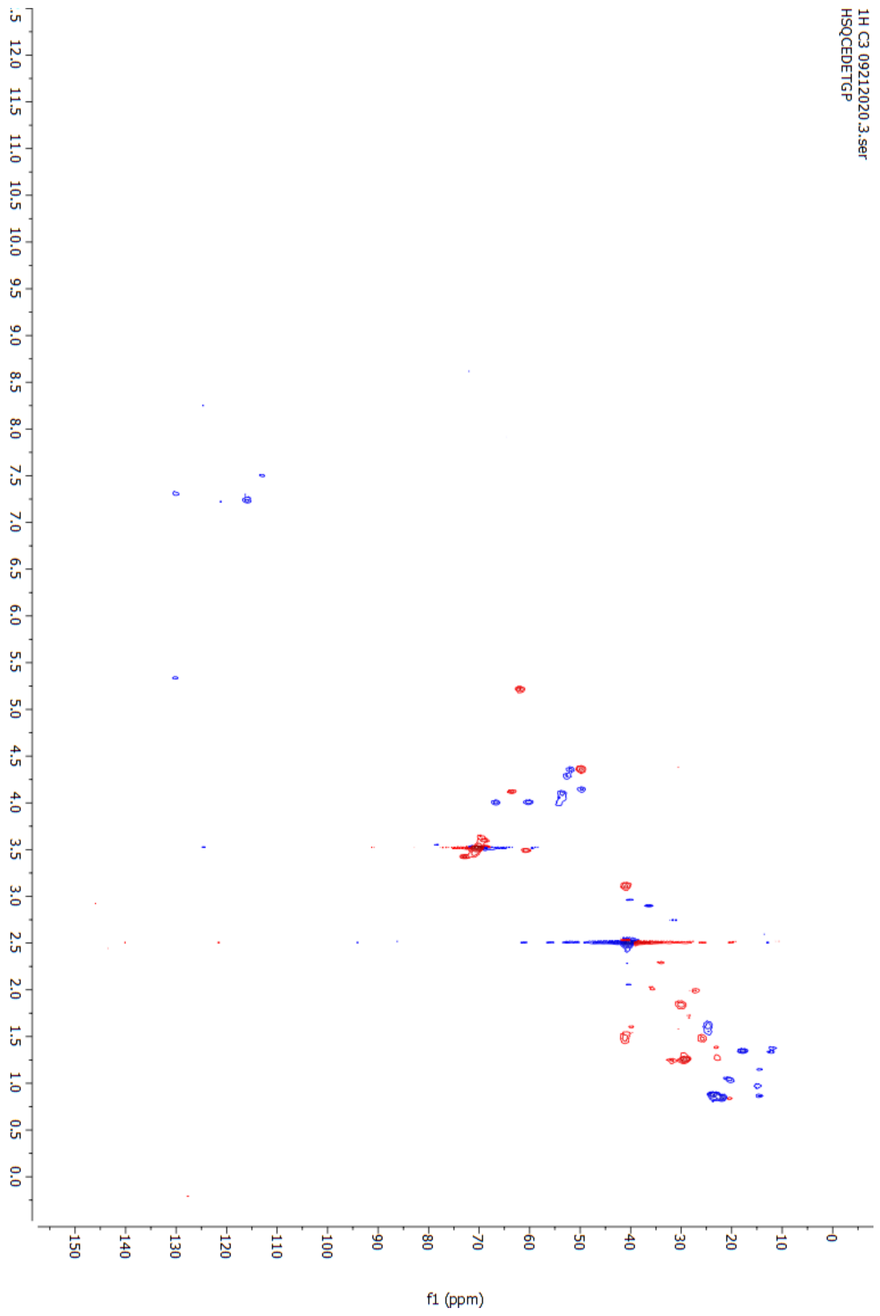

Figure S18. HSQC NMR of conjugate **5**.

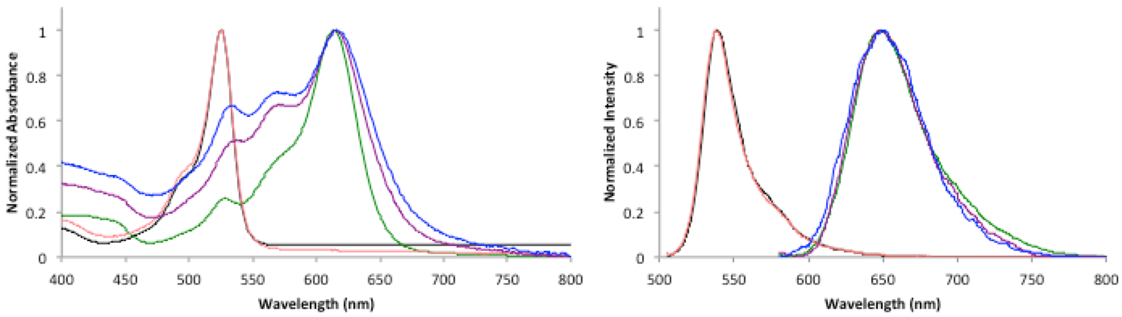

Figure S19. Normalized UV-Vis (left) and fluorescence (right) spectra for **1** (black), **2** (green), **3** (pink), **4** (purple) and **5** (blue).

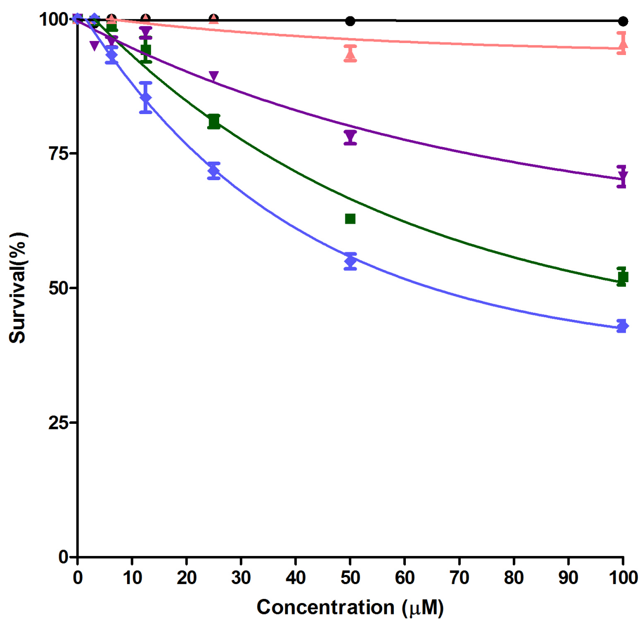

Figure S20. Phototoxicity results for **1** (black), **2** (green), **3** (pink), **4** (purple) and **5** (blue) in human carcinoma HEp2 cells.
